# Gastroesophageal reflux disease and risk of atrial fibrillation/flutter: Implications for heart failure progression

**DOI:** 10.1002/ehf2.70009

**Published:** 2025-11-09

**Authors:** Wansong Hu, Yingxing Wu, Wanqian Yu, Ping Li

**Affiliations:** ^1^ Department of Cardiovascular Medicine, The Second Affiliated Hospital, Jiangxi Medical College Nanchang University Nanchang China

**Keywords:** atrial fibrillation, atrial flutter, bidirectional, gastroesophageal reflux disease, heart failure, Mendelian randomization

## Abstract

**Aims:**

While observational studies suggest an association between gastroesophageal reflux disease (GERD) and atrial fibrillation/flutter (AF/AFL), the causal relationship and mechanisms remain undefined. This study employed Mendelian randomization (MR) to assess bidirectional causal relationships and explore potential implications for heart failure (HF) risk.

**Methods and results:**

A bidirectional two‐sample MR analysis was conducted using genome‐wide association study (GWAS) summary data from European populations (GERD: 129,080 cases and 473,524 controls; AF/AFL: 22,068 cases and 116,926 controls; Obesity: 4793 cases and 209,884 controls). Genetic instruments were selected for GERD and AF/AFL, with inverse variance weighting (IVW) as the primary analytical method to examine causality. Multivariable MR (MVMR) adjusted for obesity was performed to assess direct causal effects.

**Results:**

IVW analysis demonstrated a significant causal effect of GERD on AF/AFL risk (OR = 1.373, 95% CI = 1.208–1.600, *P* = 0.017), which persisted after MVMR adjustment for obesity (OR = 1.303, 95% CI = 1.127–1.507, *P* < 0.001). Two‐way analysis indicated no reverse causality. Sensitivity analyses supported result robustness with minimal pleiotropy.

**Conclusions:**

Genetic liability to GERD independently increases AF/AFL risk, unaffected by obesity pathways. Considering the well‐established role of AF/AFL in the pathogenesis and progression of HF, our findings position GERD as a potential modifiable target within the causal pathway. Identification and management of GERD may therefore contribute to reducing atrial arrhythmia burden and subsequent HF risk.

## Introduction

Gastroesophageal reflux disease (GERD) is a chronic condition characterized by pathological reflux of gastric contents, causing symptom burden and complications ranging from erosive esophagitis to Barrett's metaplasia.^1^ With a global prevalence exceeding 20% in adults, its epidemiological distribution exhibits marked geographical variation, influenced by lifestyle factors, obesity trends, and *Helicobacter pylori* infection patterns.^2^ Notably, rising incidence among younger populations has been attributed to dietary shifts and sedentary lifestyles. Beyond oesophageal complications (e.g., adenocarcinoma and strictures), GERD manifests extra‐oesophageal syndromes including chronic cough, laryngopharyngeal reflux, and notably, cardiac arrhythmias.^3^ The impact of GERD on health‐related quality of life is severe and comparable to the effects seen in patients with congestive heart failure (HF).^4^ Emerging evidence implicates vagal nerve activation via oesophageal irritation as a potential trigger for arrhythmic events.^5^ The substantial healthcare costs associated with GERD management underscore its socioeconomic impact. Critically, the arrhythmogenic potential of GERD extends beyond immediate rhythm disturbances to long‐term cardiac sequelae. Atrial tachyarrhythmias are established precursors to HF development, creating a pathogenic continuum where GERD may indirectly fuel the HF epidemic through arrhythmia induction.

Atrial fibrillation/flutter (AF/AFL) represents prevalent supraventricular tachyarrhythmias affecting 1–2% of the global population.^6^ These rhythm disorders share clinical manifestations (palpitations, exertional intolerance) and complications (thromboembolism, tachycardia‐induced cardiomyopathy), necessitating aggressive rhythm control through pharmacotherapy or catheter ablation.^7^ The impact of AF/AFL on patients' quality of life and overall health is significant, representing a substantial public health burden.^8^ These arrhythmias are not merely isolated incidents but important drivers of HF progression. It is estimated that 59.7 million people worldwide have AF and 64 million have HF, with the two conditions closely intertwined in their clinical presentation.^9^ Studies have shown that AF increases the risk of HF (HR = 3.4), with HFpEF being the most common phenotype.^10^ Persistent arrhythmias accelerate ventricular remodelling through tachycardia‐mediated cardiomyopathy and neuroendocrine dysfunction.

The anatomical proximity between the oesophagus and left atrium, coupled with shared autonomic innervation, provides a plausible biological basis for GERD‐AF/AFL interactions. Observational studies report bidirectional associations, yet confounding by obesity and reverse causation cloud interpretation.^11^ Current evidence remains contradictory regarding whether GERD independently increases arrhythmia risk or merely represents an epiphenomenon of shared pathophysiology. Importantly, the GERD‐AF/AFL relationship acquires added clinical significance when viewed through the lens of HF prevention, as early interception of modifiable arrhythmia triggers could potentially attenuate downstream heart failure incidence.

Mendelian randomization (MR) is a widely used method that improves causal inference by using genetic variants randomly assigned during meiosis as instrumental variables. This approach is less likely to be influenced by disease state and environment, thus reducing the impact of unobserved confounders.^12^ In this study, we conducted a two‐sample pooled data MR analysis to investigate the bidirectional association between GERD and AF/AFL. By establishing this causal relationship, we hope to identify a new modifiable target to interrupt the AF‐HF cascade. Additionally, given the robust association between obesity and GERD, as well as previous observational and MR studies suggesting a potential causal link between obesity and AF,^13^, ^14^, ^15^, ^16^, ^17^ a multivariate MR analysis was conducted to investigate the direct impact of GERD on AF/AFL while accounting for the influence of obesity.

## Methods

### Data sources

The relevant genome‐wide association study datasets were obtained from the IEU (Integrative Epidemiology Unit) OpenGWAS (Genome‐wide association studies) (https://gwas.mrcieu.ac.uk). The GWAS dataset for GERD was obtained from the European Bioinformatics Centre (EBIC), which consisted of 129,080 patients of European origin and 473,524 controls. Similarly, the GWAS dataset for atrial fibrillation and atrial flutter was obtained from the FinnGen consortium, comprising 22,068 patients of European origin and 116,926 controls, and summary GWAS data for obesity including 4793 cases and 209,884 controls (*Table* *S1*).

### Selection of instrumental variables

Significant SNPs (Single Nucleotide Polymorphism) associated with exposure (*P* < 5.0 × 10^−8^) were selected, and linkage disequilibrium was excluded to ensure that SNPs were independent of each other (r^2^ < 0.001, genetic distance of 10,000 kb).^18^ The corresponding data for instrumental variables (IV) in the combined exposure and outcome, including chromosomal location information, effect allele, allele effect size (Beta), standard error, and *P*‐values from the pooled GWAS data of the exposure and outcome, were obtained. All MR analyses were performed on the MR Basis platform. We assessed the presence of weak IV bias in the selected instrumental variables by calculating the *F* statistic, which was calculated using the following formula: *F* = R^2^(N − 2)/(1 − R^2^), where R^2^ represents the proportion of the variation in exposure coefficients that is explained by each IV, and N is the sample size of the dataset. We calculated the R^2^ using the following formula: R^2^ = 2 × EAF × (1 − EAF) × β^2^/[2 × EAF × (1 − EAF) × β^2^ + 2 × EAF × (1 − EAF) × N × se(β)^2^].^19^ If *F* > 10, it indicates no weak IVs bias.^20^

### Statistical analyses

**Figure 1:**
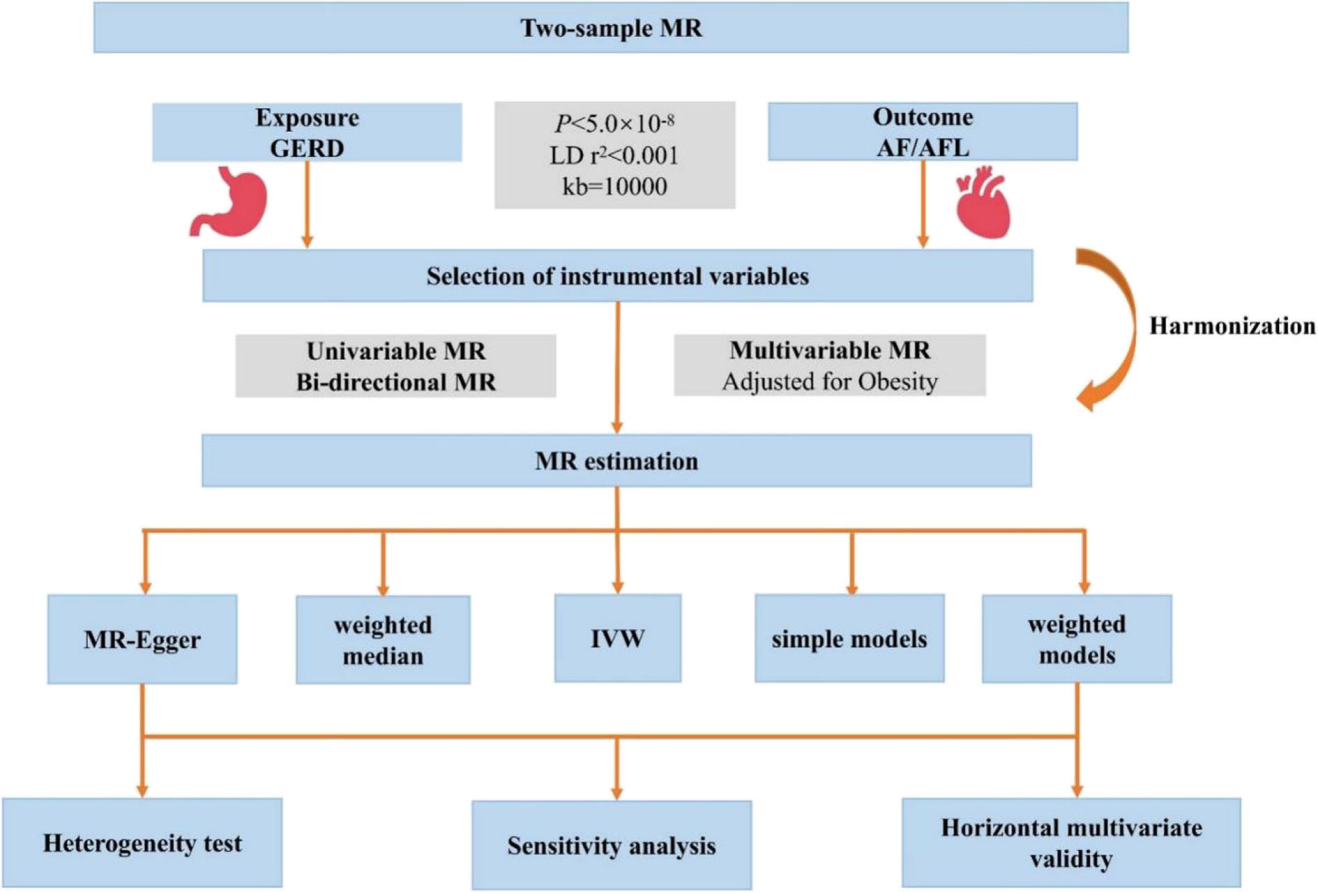
The flow diagram of the Mendelian randomization (MR) study. AF, atrial fibrillation; AFL, atrial flutter; GERD, gastroesophageal reflux disease; MR, Mendelian randomization.

The overall design of this study can be seen in *Figure* *1*. The two‐sample MR analyses in this study utilized inverse variance weighting (IVW), weighted median, MR‐Egger regression, simple mode, and weighted mode. MR‐Egger was used to test for horizontal multivariate validity, and the results indicated that there was no statistically significant difference between the intercept and 0, suggesting no horizontal multivariate validity. This means that the instrumental variable solely affects the outcome through exposure. The main findings of this study were based on the IVW method, which combines the estimation of Wald ratios for the causal effects of different SNPs.^21^ The results obtained from other methods were used as sensitivity analyses. To enhance result interpretation, the Beta values were converted to Odds Ratios (OR), and 95% Confidence Intervals (CI) were calculated. The study employed the MR Polytropic Residuals and Outliers (MR‐PRESSO) method to assess and correct for horizontal polytropy.^22^ Additionally, a ‘leave‐one‐out’ analysis was conducted, sequentially excluding one SNP at a time and calculating the Mendelian randomization effect of the remaining SNPs. The results were presented in a forest plot, allowing for visual assessment of each SNP's effect on the results and determining the stability of the TSMR analysis. Leave‐one‐out analyses were also performed to evaluate if a single SNP was driving the MR estimates. The statistical power of MR (power > 80%) was calculated via the mRnd tool on the website (https://shiny.cnsgenomics.com/).

Besides, as previous studies suggest a strong association between obesity and AF/AFL, we performed multivariable Mendelian randomization adjusting for this conformer to show a direct causal effect of GERD on AF/AFL. The flow schematic of this study is shown in *Figure* *1*. The statistical analyses in this study were conducted using R (version 4.3.1) and the R package ‘TwoSampleMR’ (0.5.7) and ‘MVMR’ (version 0.4). A significance level of α = 0.05 (*P* < 0.05) was used to determine statistical significance.

## Results

### Causal effect of GERD on AF/AFL

#### SNPs: Basic information

**Figure 2:**
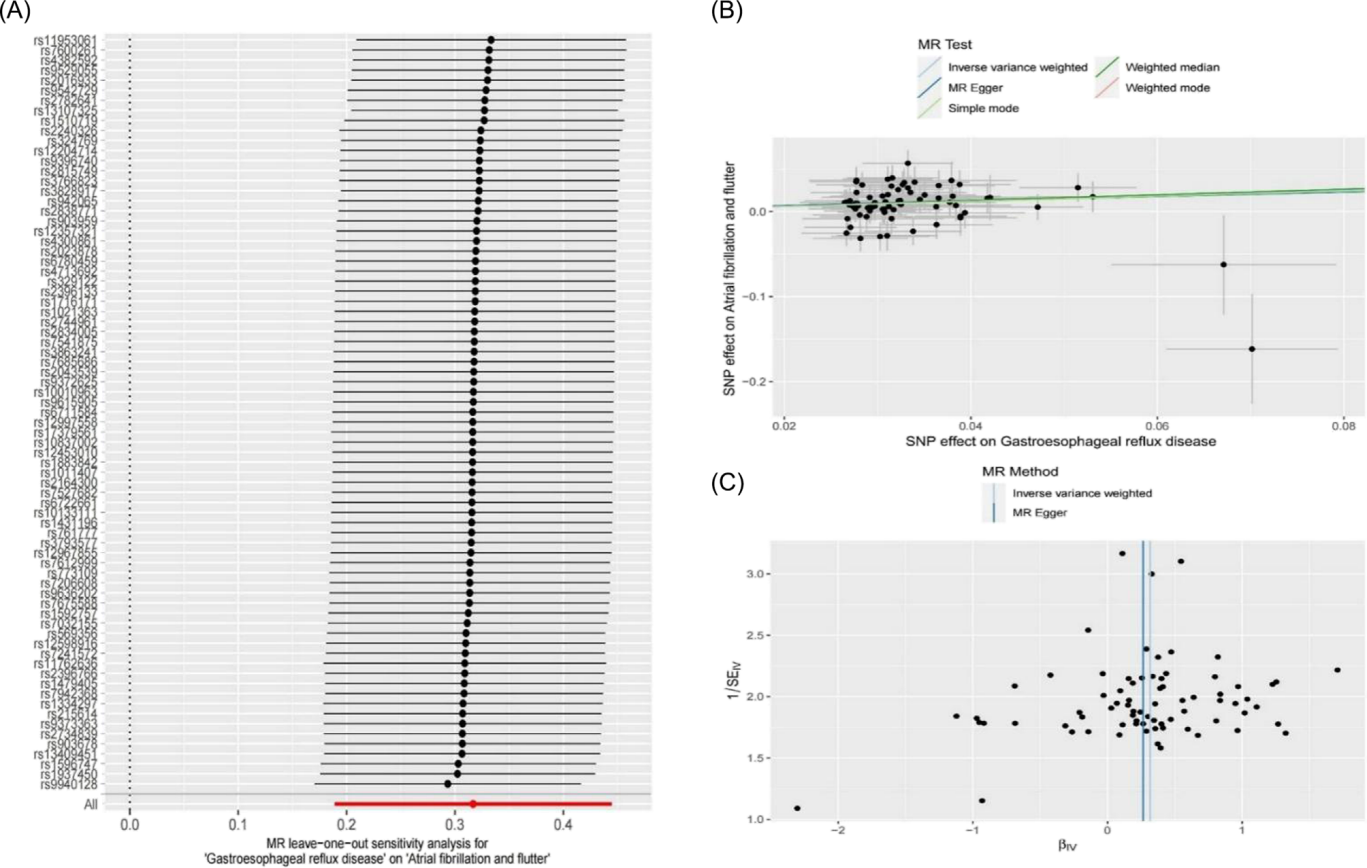
Forest and leave‐one‐out plot for IVW MR of GERD on AF/AFL (A), sensitivity analysis (B), and the funnel plot from genetically predicted GERD on AF/AFL.

GERD was considered the exposure factor, while AF/AFL was the outcome variable. A total of 75 SNPs were screened and identified as instrumental variables (IVs) with an *F*‐value > 10 (*Table* *S2*). The intercept of the MR‐Egger regression can be used as an indicator to assess whether the horizontal multiplicity of IVs affects the results of the TSMR analysis. The intercept value is close to 0 (intercept = 0.002, *P* = 0.891) (*Table* *1*), suggesting that there is no horizontal pleiotropy among the instrumental variables (*Figure* *2* *B*).

**Table 1:** Heterogeneity test and horizontal pleiotropy test.

Exposure	Outcome	Heterogeneity test (MR‐Egger)			Heterogeneity test (IVW)			Horizontal pleiotropy test (MR‐Egger)	
		Cochran's *Q*	Q_df	*P*	Cochran's Q	Q_df	*P*	Intercept	*P*
GERD	AF	93.47	73	0.054	93.49	74	0.063	0.002	0.891
AF	GERD	33.30	17	0.010	33.32	18	0.015	0.0003	0.933

IVW, inverse variance weighting; MR, Mendelian randomization.

#### Two‐sample Mendelian randomization analysis

The Mendelian randomization analyses provide evidence supporting a causal relationship between genetic susceptibility to GERD and an increased risk of AF/AFL. The statistical power of the MR analysis was estimated to be approximately 100%. In this study, the IVW method was used as the primary approach to assess the causal relationship between genetic susceptibility to GERD and the increased risk of AF/AFL. The IVW results showed an odds ratio (OR) of 1.373, with a 95% confidence interval (CI) of 1.208 to 1.600, and a *P*‐value of 1.174 × 10^–6^. Results from other methods, including MR‐Egger (OR = 1.303, 95% CI: 0.611 to 2.778, *P* = 0.496), weighted median (OR = 1.389, 95% CI: 1.179 to 1.637, *P* = 8.446e‐05), simple model (OR = 1.349, 95% CI: 0.928 to 1.963, *P* = 0.121), and weighted model (OR = 1.349, 95% CI: 0.961 to 1.896, *P* = 0.088), are presented in *Table* *2*.

**Table 2:** Mendelian randomization analysis of causal association between GERD and the risk of AF.

Methods	SNPs	Beta	SE	OR (95% CI)	*P*
MR‐Egger	75	0.265	0.386	1.303 (0.611–2.778)	0.496
Weighted median	75	0.329	0.084	1.389 (1.179–1.637)	8.446 × 10^−5^
IVW	75	0.317	0.065	1.373 (1.208–1.600)	1.174 × 10^−6^
Simple mode	75	0.300	0.191	1.349 (0.928–1.963)	0.121
Weighted mode	75	0.300	0.173	1.349 (0.961–1.896)	0.088

AF, atrial fibrillation; CI, confidence interval; GERD, gastroesophageal reflux disease; IVW, inverse variance weighting; MR, Mendelian randomization; OR, odds ratios; SE, standard error; SNP, single nucleotide polymorphism.

#### Heterogeneity test and sensitivity analysis

IVW and MR‐Egger regression analyses were conducted to identify heterogeneity among instrumental variables. Cochran's *Q* test was used to quantify heterogeneity, with *P* < 0.05 indicating significant heterogeneity. If heterogeneity was present, a random effects IVW model was used to estimate causal effects. Both MR‐Egger regression (Cochran's *Q* = 93.47, *P* = 0.054) and IVW (Cochran's *Q* = 93.49, *P* = 0.063) analyses (*Table* *1*) indicated no heterogeneity among IVs (*Table* *1*, *Figure* *2* *B*).

Sensitivity analyses were performed using the leave‐one‐out method, where SNPs were removed one by one to compare the causal effects of the remaining SNPs with the results of the TSMR analyses of all SNPs. This was done to determine if the causal associations originated from a single IV. The MR‐PRESSO test also was used to identify the outlier SNPs (*Table* *S3*). The results are presented in a forest plot, demonstrating the robustness of the TSMR analyses (*Figure* *2* *A*).

#### Reverse TSMR analysis

**Figure 3:**
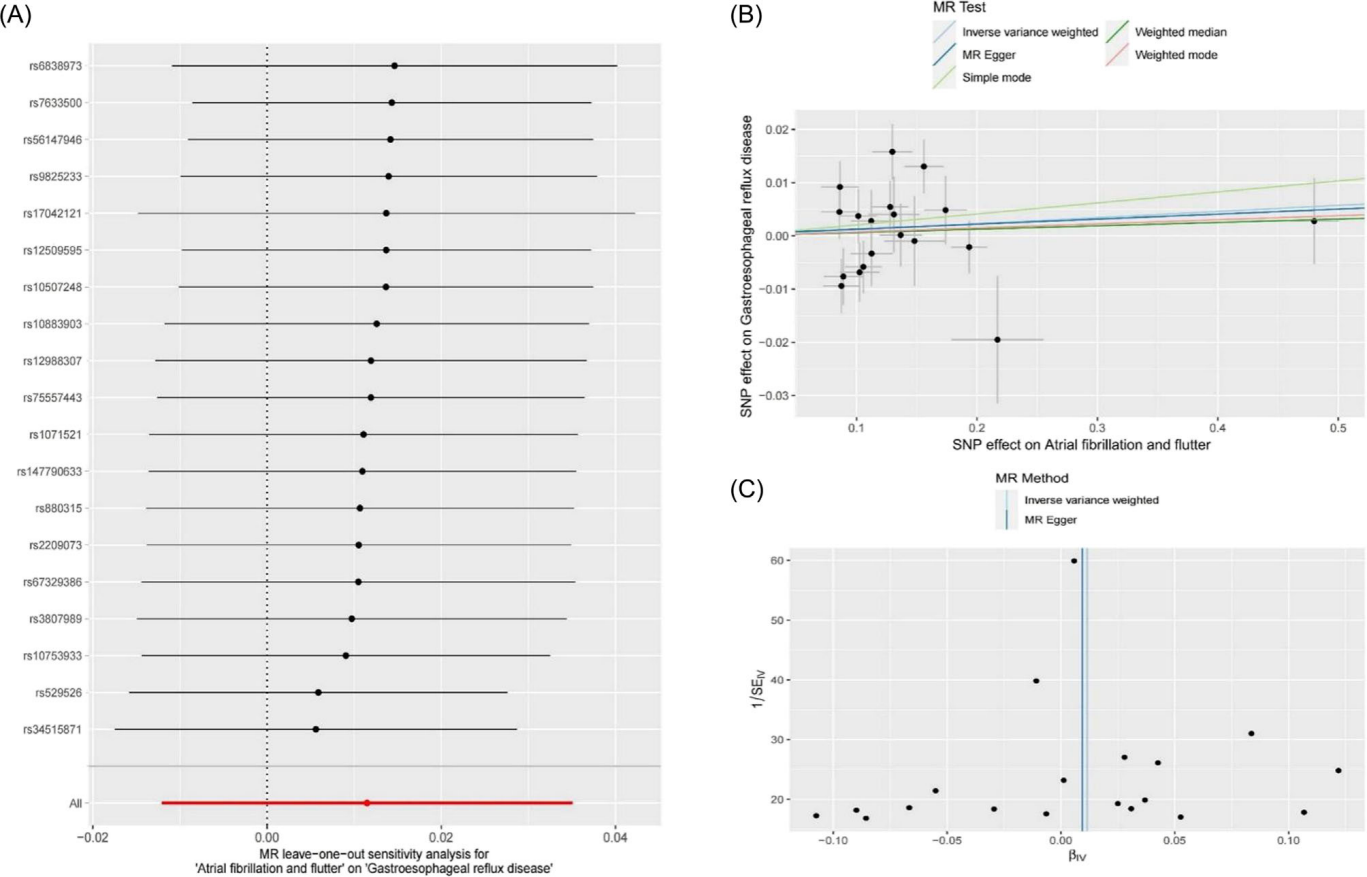
Forest and leave‐one‐out plot for IVW MR of AF/AFL on GERD (A), sensitivity analysis (B), and the funnel plot from genetically predicted AF/AFL on GERD.

In this study, we utilized reverse two‐sample Mendelian randomization (TSMR) to investigate the potential causal relationship between genetic susceptibility to AF/AFL and the risk of GERD. We screened a total of 19 single nucleotide polymorphisms (SNPs) and identified them as instrumental variables (*Table* *S4*). The *F*‐value was found to be greater than 10, indicating strong instrument strength. The test for horizontal pleiotropy suggested that there was no evidence of horizontal pleiotropy for the screened instrumental variables, as indicated by the MR‐Egger regression intercept of 0.0003 (*P* = 0.933) (*Table* *1*). The results of the Mendelian randomization analysis did not support a causal relationship between genetic susceptibility to AF/AFL and an increased risk of GERD. The Inverse Variance Weighted (IVW) method yielded an odds ratio (OR) of 1.012 (95% CI: 0.988–1.036, *P* = 0.340), suggesting no significant association. Consistent results were obtained from other methods, including MR‐Egger (OR = 1.009, 95% CI: 0.957–1.065, *P* = 0.736), weighted median (OR = 1.006, 95% CI: 0.980–1.033, *P* = 0.640), simple model (OR = 1.021, 95% CI: 0.975–1.069, *P* = 0.386), and weighted mode (OR = 1.008, 95% CI: 0.979–1.037, *P* = 0.615) (*Table* *3*, *Figure* *3* *B*). Furthermore, the heterogeneity test indicated relatively small heterogeneity for both MR‐Egger regression (Cochran's *Q* = 33.30, *P* = 0.010) and IVW (Cochran's *Q* = 33.32, *P* = 0.015) (*Table* *1*, *Figure* *3* *C*). The MR‐PRESSO analysis also suggested a small degree of heterogeneity (RSSobs = 36.42, *P* = 0.027) (*Table* *S3*), but no significant outliers were identified. Sensitivity analyses using the leave‐one‐out method confirmed the reliability of the TSMR results (*Figure* *3* *A*).

**Table 3:** Mendelian randomization analysis of causal association between AF and the risk of GERD.

Methods	SNPs	Beta	SE	OR (95% CI)	*P*
MR‐Egger	19	0.009	0.027	1.009 (0.957–1.065)	0.736
Weighted median	19	0.006	0.014	1.006 (0.980–1.033)	0.640
IVW	19	0.011	0.012	1.012 (0.988–1.036)	0.340
Simple mode	19	0.021	0.023	1.021 (0.975–1.069)	0.386
Weighted mode	19	0.008	0.015	1.008 (0.979–1.037)	0.615

AF, atrial fibrillation; CI, confidence interval; GERD, gastroesophageal reflux disease; IVW, inverse variance weighting; MR, Mendelian randomization; OR, odds ratios; SE, standard error; SNP, single nucleotide polymorphism.

#### Multivariable Mendelian randomization analysis

Because of the potential influence of obesity, multivariate Mendelian randomization analysis was conducted to assess the direct causal effect of GERD on AF/AFL. There were 158 independent SNPs selected as instrumental variables for GERD and obesity. After adjusting for this potential confounder, GERD still showed a direct effect on AF/AFL (OR, 1.303; 95% CI, 1.127–1.507; *P* < 0.001) (*Table* *4*). Moreover, in sensitivity analyses, multivariable MR‐Egger regression suggested that there was no horizontal pleiotropy (Intercept *P* = 0.090) or heterogeneity (*P* = 0.074) in MR analysis (*Table* *4*).

**Table 4:** Results of multivariable MR analysis.

Exposure	Outcome	Method	OR	95% CI	*P*
GERD	AF/AFL	MR‐Egger	1.480	0.716–2.069	0.256
Obesity			1.083	0.984–1.176	0.101
GERD	AF/AFL	IVW	1.303	1.127–1.507	<0.001
Obesity			1.080	0.983–1.186	0.108
GERD	AF/AFL	Median	1.340	1.110–1.476	0.002
Obesity			1.061	0.919–1.202	0.404

AF, atrial fibrillation; AFL, atrial flutter; CI, confidence interval; GERD, gastroesophageal reflux disease; IVW, inverse variance weighting; MR, Mendelian randomization; OR, odds ratios.

## Discussion

In this study, we conducted an analysis of the pooled GWAS dataset using a bidirectional TSMR approach. We found that there is a causal relationship between genetic susceptibility to GERD and an increased risk of AF/AFL. The instrumental variable weighted (IVW) method yielded an odds ratio (OR) of 1.373 (95% CI: 1.208–1.600, *P* = 1.174 × 10^−6^) in support of this relationship. However, our findings did not provide evidence for a causal relationship between genetic susceptibility to AF/AFL and an increased risk of GERD. The IVW method yielded an OR of 1.012 (95% CI: 0.988–1.036, *P* = 0.340) for this relationship. Sensitivity analyses confirmed the reliability of our Mendelian Randomization results.

Besides, there is mounting evidence suggesting a connection between obesity and an increased risk of AF/AFL. Given that obesity is linked to GERD,^23^, ^24^ it is important to consider the potential impact of obesity when investigating the relationship between GERD and AF/AFL. Our multivariate MR analysis included GERD and obesity to determine if the influence of GERD on AF/AFL was independent of obesity. The results of our study, specifically the IVW analysis, indicated that GERD was still correlated with a heightened risk of AF/AFL even after adjusting for obesity. This suggests that the causal effect of GERD on AF/AFL persists even when accounting for the influence of obesity. On the other hand, some traditional risk factors for AF/AFL should not be overlooked. Hypertension is a major modifiable risk factor, driving atrial remodelling through pressure overload and neurohormonal activation, while chronic kidney disease increases the risk of AF/AFL through uremic cardiomyopathy and electrolyte imbalance.^25^ Age‐related myocardial cell aging and male gender further exacerbate the risk.^26^ Crucially, our multivariate MR results suggest that GERD may act through independent mechanisms. However, this independence does not rule out clinical synergistic effects, and GERD management should be combined with conventional risk stratification, particularly in elderly or hypertensive cohorts requiring enhanced monitoring.

A number of prior observational studies proposed a link between GERD and AF/AFL; however, until now, it remained unclear whether this relationship is causal and in what direction these two disease entities were related to each other. Ludwig Roemheld et al. first described the link between gastrointestinal symptoms and cardiac arrhythmias and named it ‘Roemheld's gastrocardiac syndrome’.^27^ Kunz et al. found that the presence of GERD increased the risk of atrial fibrillation to 40%, even after excluding common cardiovascular risk factors.^28^ In a study of three patients, atrial fibrillation was found to be associated with a decrease in pH during 24‐hour oesophageal pH monitoring.^29^ Possible explanations for this association include local and systemic inflammation caused by acid reflux. In addition, another large population‐based survey study in Taiwan also demonstrated an independent association between GERD and an increased risk of AF (HR 1.31; 95% CI 1.06–1.61, *P* = 0.013).^30^ It has also been suggested that while GERD may not be a risk factor for the development of AF, it is associated with AF recurrence after radiofrequency ablation.^31^

In contrast, some prospective and retrospective studies have found a negative correlation between GERD and AF, and the presence of AF seems to increase the incidence of GERD.^32^, ^33^ Several multicenter studies have also shown a significant association between AF and symptomatic GERD.^34^, ^35^ A meta‐analysis conducted by Lu et al. consisting of seven observational studies revealed that the adjusted overall relative risk (RR) for GERD induced by AF and AF induced by GERD was 1.54 (95% CI, 1.08–2.17) and 1.06 (95% CI, 0.86–1.31), respectively, suggesting that AF is associated with an increased risk of GERD.^36^ It is speculated that an enlarged and fibrillating left atrium may compress or irritate the adjacent lower oesophagus. Additionally, it has been observed that a significant number of patients undergoing radiofrequency ablation of AF develop pathological acid reflux after the procedure,^37^, ^38^ which may be attributed to damage to the oesophageal wall^39^ and impaired oesophageal dynamics resulting from the ablation therapy.^40^ Therefore, the routine use of proton pump inhibitors in patients undergoing catheter ablation for atrial fibrillation may be necessary. However, there are also studies that have reported the opposite result, finding that typical symptoms of gastroesophageal reflux disease are uncommon after AF ablation,^41^ and that oesophageal damage is relatively mild.^42^ The study by Coutinho et al., on the other hand, concluded that the correlation between reflux episodes and cardiac arrhythmias was low, and the results did not support a causal link between the two.^43^ Nevertheless, there are some studies that do not support an association between AF and GERD, which remains somewhat controversial.

However, this study has several limitations. Firstly, the MR results were based on a European population, and further studies are required to determine if a causal relationship exists in other populations. Secondly, the SNPs used for analysis may be associated with other traits due to genetic polymorphisms. Additionally, the outcome variables in this study focused on AF and atrial flutter, and it may be necessary to assess the difference in the effect of exposure on these two conditions through more studies, despite their clinical similarities and frequent coexistence.

In conclusion, this is the first MR study to explore the causal effect of GERD on AF/AFL, which also carries inherent significance for HF prevention. Bidirectional TSMR analyses provide evidence supporting a causal association between genetic susceptibility to GERD and an increased risk of AF/AFL, and the effect persisted even when we accounted for the impact of obesity. However, there is currently no evidence supporting a causal association between genetic susceptibility to AF/AFL and an increased risk of GERD. Nevertheless, further studies are required to confirm these findings due to the limitations of the current studies. Understanding the relationship between acid reflux disease and AF/AFL is crucial for developing comprehensive treatment strategies that can improve the prognosis of AF/AFL patients. The 37.3% increased risk of AF observed in genetically susceptible GERD patients may translate into measurable HF incidence. Given the high prevalence of GERD in the population and its modifiability, our findings advocate for enhanced arrhythmia monitoring, such as 12‐lead electrocardiogram (ECG) testing. For individuals with additional risk factors or significant nocturnal reflux and extraesophageal symptoms, stratified monitoring should be considered, and 24‐hour Holter monitoring may be necessary. Moderate reduction of AF risk through reflux management may also alleviate the burden of HF. Additionally, early initiation of PPIs for GERD may offer a potential approach to reducing the risk of developing AF/AFL. However, more prospective studies are needed to evaluate the effectiveness of this approach.

## Funding

This work was supported by the National Natural Science Foundation of China (grant nos. 82460079). The funding body had no role in the design of the study and the collection, analysis, and interpretation of data and in writing the manuscript.

## Conflict of Interest

The authors have no relevant financial or non‐financial interests to disclose.

## Supporting information


**Table S1.** Descriptive details about exposure dataset and outcome dataset.

**Table S2.** Summary statistics for the instrumental variables associated with GERD and AF/AFL.

**Table S3.** MR‐PRESSO test of causal association between Exposure and Outcome.

**Table S4.** Summary statistics for the instrumental variables associated with AF/AFL and GERD.

## Data Availability

The relevant genome‐wide association study datasets were obtained from the IEU OpenGWAS project (https://gwas.mrcieu.ac.uk).

## References

[1] Fass R. . Gastroesophageal reflux disease. N Engl J Med 2022;387(13):1207–1216. doi:10.1056/NEJMcp2114026 36170502

[2] Eusebi LH , Ratnakumaran R , Yuan Y , Solaymani‐Dodaran M , Bazzoli F , Ford AC . Global prevalence of, and risk factors for, gastro‐oesophageal reflux symptoms: a meta‐analysis. Gut 2018;67(3):430–440. doi:10.1136/gutjnl-2016-313589 28232473

[3] Vaezi MF , Katzka D , Zerbib F . Extraesophageal symptoms and diseases attributed to GERD: where is the pendulum swinging now?. Clin Gastroenterol Hepatol 2018;16(7):1018–1029. doi:10.1016/j.cgh.2018.02.001 29427733

[4] Liu H‐F , Zhang J‐G , Li J , Chen X‐G , Wang W‐A . Improvement of clinical parameters in patients with gastroesophageal reflux disease after radiofrequency energy delivery. World J Gastroenterol 2011;17(39):4429–4433. doi:10.3748/wjg.v17.i39.4429 22110270 PMC3218158

[5] Reddy YM , Singh D , Nagarajan D , Pillarisetti J , Biria M , Boolani H , Emert M , Chikkam V , Ryschon K , Vacek J , Bommana S , Atkins D , Verma A , Olyaee M , Dawn B , Lakkireddy D . Atrial fibrillation ablation in patients with gastroesophageal reflux disease or irritable bowel syndrome‐the heart to gut connection!. J Interv Card Electrophysiol 2013;37(3):259–265. doi:10.1007/s10840-013-9807-5 23736874

[6] Brundel BJJM , Ai X , Hills MT , Kuipers MF , Lip GYH de Groot NMS . Atrial fibrillation. Nat Rev Dis Primers 2022;8(1):21. doi:10.1038/s41572-022-00347-9 35393446

[7] Calkins H . Important differences exist between atrial fibrillation and atrial flutter in atrial remodeling. J Am Coll Cardiol 2020;76(4):389–390. doi:10.1016/j.jacc.2020.06.004 32703508

[8] Dong X‐J , Wang B‐B , Hou F‐F , Jiao Y , Li H‐W , Lv S‐P , Li FH . Global burden of atrial fibrillation/atrial flutter and its attributable risk factors from 1990 to 2019. Europace 2023;25(3):793–803. doi:10.1093/europace/euac237 36603845 PMC10062373

[9] Savarese G , Becher PM , Lund LH , Seferovic P , Rosano GMC , Coats AJS . Global burden of heart failure: a comprehensive and updated review of epidemiology. Cardiovasc Res 2023;118(17):3272–3287. doi:10.1093/cvr/cvac013 35150240

[10] Stewart S , Hart CL , Hole DJ , McMurray JJV . A population‐based study of the long‐term risks associated with atrial fibrillation: 20‐year follow‐up of the Renfrew/Paisley study. Am J Med 2002;113(5):359–364. 12401529 10.1016/s0002-9343(02)01236-6

[11] Chen Y . Gastroesophageal reflux disease and non‐digestive tract diseases. Expert Rev Gastroenterol Hepatol 2015;9(5):685–692. doi:10.1586/17474124.2015.1012495 25665699

[12] Sekula P , Del Greco MF , Pattaro C , Köttgen A . Mendelian randomization as an approach to assess causality using observational data. J Am Soc Nephrol 2016;27(11):3253–3265. 27486138 10.1681/ASN.2016010098 PMC5084898

[13] Frost L , Hune LJ , Vestergaard P . Overweight and obesity as risk factors for atrial fibrillation or flutter: the Danish diet, cancer, and health study. Am J Med 2005;118(5):489–495. 15866251 10.1016/j.amjmed.2005.01.031

[14] Chatterjee NA , Giulianini F , Geelhoed B , Lunetta KL , Misialek JR , Niemeijer MN , Rienstra M , Rose LM , Smith AV , Arking DE , Ellinor PT , Heeringa J , Lin H , Lubitz SA , Soliman EZ , Verweij N , Alonso A , Benjamin EJ , Gudnason V , Stricker BHC , van der Harst P , Chasman DI , Albert CM . Genetic obesity and the risk of atrial fibrillation: causal estimates from Mendelian randomization. Circulation 2017;135(8):741–754. doi:10.1161/CIRCULATIONAHA.116.024921 27974350 PMC5322057

[15] Ardissino M , Reddy RK , Slob EAW , Patel KHK , Ryan DK , Gill D , Ng FS . Sleep disordered breathing, obesity and atrial fibrillation: a Mendelian randomisation study. Genes (Basel) 2022;13(1):104. doi:10.3390/genes13010104 35052444 PMC8774383

[16] Ma, M , Zhi, H , Yang, S , Yu, EY‐W , Wang, L . Body mass index and the risk of atrial fibrillation: a Mendelian randomization study. Nutrients 2022;14(9). doi:10.3390/nu14091878 PMC9101688 35565843

[17] Sha R , Baines O , Hayes A , Tompkins K , Kalla M , Holmes AP , O'Shea C , Pavlovic D . Impact of obesity on atrial fibrillation pathogenesis and treatment options. J Am Heart Assoc 2024;13(1):e032277. doi:10.1161/JAHA.123.032277 38156451 PMC10863823

[18] Burgess S , Butterworth A , Thompson SG . Mendelian randomization analysis with multiple genetic variants using summarized data. Genet Epidemiol 2013;37(7):658–665. doi:10.1002/gepi.21758 24114802 PMC4377079

[19] He B , Lyu Q , Yin L , Zhang M , Quan Z , Ou Y . Depression and osteoporosis: a Mendelian randomization study. Calcif Tissue Int 2021;109(6):675–684. doi:10.1007/s00223-021-00886-5 34259888 PMC8531056

[20] Hemani G , Zheng J , Elsworth B , Wade KH , Haberland V , Baird D , Laurin C , Burgess S , Bowden J , Langdon R , Tan VY , Yarmolinsky J , Shihab HA , Timpson NJ , Evans DM , Relton C , Martin RM , Davey Smith G , Gaunt TR , Haycock PC . The MR‐base platform supports systematic causal inference across the human phenome. Elife 2018;7:7. doi:10.7554/eLife.34408 PMC5976434 29846171

[21] Bowden J , Davey Smith G , Burgess S . Mendelian randomization with invalid instruments: effect estimation and bias detection through egger regression. Int J Epidemiol 2015;44(2):512–525. doi:10.1093/ije/dyv080 26050253 PMC4469799

[22] Ong J‐S , MacGregor S . Implementing MR‐PRESSO and GCTA‐GSMR for pleiotropy assessment in Mendelian randomization studies from a practitioner's perspective. Genet Epidemiol 2019;43(6):609–616. doi:10.1002/gepi.22207 31045282 PMC6767464

[23] Yuan S , Larsson SC . Adiposity, diabetes, lifestyle factors and risk of gastroesophageal reflux disease: a Mendelian randomization study. Eur J Epidemiol 2022;37(7):747–754. doi:10.1007/s10654-022-00842-z 35119566 PMC9329382

[24] Shay JES , Singh A . The effect of obesity on gastrointestinal disease. Gastroenterol Clin North Am 2023;52(2):403–415. doi:10.1016/j.gtc.2023.03.008 37197882

[25] Alonso A , Lopez FL , Matsushita K , Loehr LR , Agarwal SK , Chen LY , Soliman EZ , Astor BC , Coresh J . Chronic kidney disease is associated with the incidence of atrial fibrillation: the atherosclerosis risk in communities (ARIC) study. Circulation 2011;123(25):2946–2953. doi:10.1161/CIRCULATIONAHA.111.020982 21646496 PMC3139978

[26] Go AS , Hylek EM , Phillips KA , Chang Y , Henault LE , Selby JV , Singer DE . Prevalence of diagnosed atrial fibrillation in adults: national implications for rhythm management and stroke prevention: the AnTicoagulation and risk factors in atrial fibrillation (ATRIA) study. JAMA 2001;285:(18):2370–2375. 11343485 10.1001/jama.285.18.2370

[27] Jervell, O , LØDØEn, O . The gastrocardiac syndrome. Acta Med Scand Suppl 1952;266:595–599. doi:10.1111/j.0954-6820.1952.tb13409.x 14902409

[28] Kunz JS , Hemann B , Edwin Atwood J , Jackson J , Wu T , Hamm C . Is there a link between gastroesophageal reflux disease and atrial fibrillation?. Clin Cardiol 2009;32(10):584–587. doi:10.1002/clc.20660 19911354 PMC6653088

[29] Gerson LB , Friday K , Triadafilopoulos G . Potential relationship between gastroesophageal reflux disease and atrial arrhythmias. J Clin Gastroenterol 2006;40:(9):828–832. 17016140 10.1097/01.mcg.0000225571.42890.a5

[30] Huang C‐C , Chan W‐L , Luo J‐C , Chen Y‐C , Chen T‐J , Chung C‐M , Huang PH , Lin SJ , Chen JW , Leu HB . Gastroesophageal reflux disease and atrial fibrillation: a nationwide population‐based study. PLoS ONE 2012;7(10):e47575. doi:10.1371/journal.pone.0047575 23077642 PMC3471851

[31] Lioni L , Letsas KP , Efremidis M , Vlachos K , Karlis D , Asvestas D , Mihas CC , Sideris A . Gastroesophageal reflux disease is a predictor of atrial fibrillation recurrence following left atrial ablation. Int J Cardiol 2015;183:211–213. doi:10.1016/j.ijcard.2015.01.083 25675903

[32] Bunch TJ , Packer DL , Jahangir A , Locke GR , Talley NJ , Gersh BJ , Roy RR , Hodge DO , Asirvatham SJ . Long‐term risk of atrial fibrillation with symptomatic gastroesophageal reflux disease and esophagitis. Am J Cardiol 2008;102(9):1207–1211. doi:10.1016/j.amjcard.2008.06.048 18940293 PMC2895499

[33] Hwang JJ , Lee DH , Yoon H , Shin CM , Park YS , Kim N . Is atrial fibrillation a risk factor for gastroesophageal reflux disease occurrence?. Medicine (Baltimore) 2015;94(43):e1921. doi:10.1097/MD.0000000000001921 26512618 PMC4985431

[34] Kubota S , Nakaji G , Shimazu H , Odashiro K , Maruyama T , Akashi K . Further assessment of atrial fibrillation as a risk factor for gastroesophageal reflux disease: a multicenter questionnaire survey. Intern Med 2013;52:(21):2401–2407. 24190143 10.2169/internalmedicine.52.0923

[35] Shimazu H , Nakaji G , Fukata M , Odashiro K , Maruyama T , Akashi K . Relationship between atrial fibrillation and gastroesophageal reflux disease: a multicenter questionnaire survey. Cardiology 2011;119(4):217–223. doi:10.1159/000331497 21985841

[36] Xu L , Zhang Y , Xie J , Liu Y , Xu L . Association between gastroesophageal reflux disease and atrial fibrillation: a systematic review and meta‐analysis. Rev Esp Enferm Dig 2019;111(11):874–879. doi:10.17235/reed.2019.5389/2017 31617365

[37] Martinek M , Hassanein S , Bencsik G , Aichinger J , Schoefl R , Bachl A , Gerstl S , Nesser HJ , Purerfellner H . Acute development of gastroesophageal reflux after radiofrequency catheter ablation of atrial fibrillation. Heart Rhythm 2009;6(10):1457–1462. doi:10.1016/j.hrthm.2009.06.022 19716773

[38] Stevenson WG , Saltzman JR . Gastroesophageal reflux and atrial‐esophageal fistula. Heart Rhythm 2009;6(10):1463–1464. doi:10.1016/j.hrthm.2009.07.023 19968925

[39] Schmidt M , Nölker G , Marschang H , Gutleben K‐J , Schibgilla V , Rittger H , et al. Incidence of oesophageal wall injury post‐pulmonary vein antrum isolation for treatment of patients with atrial fibrillation. Europace 2008;10(2):205–209. doi:10.1093/europace/eun001 18256125

[40] Tolone S , Savarino E , Docimo L . Radiofrequency catheter ablation for atrial fibrillation elicited ‘jackhammer esophagus’: a new complication due to vagal nerve stimulation?. J Neurogastroenterol Motil 2015;21(4):612–615. doi:10.5056/jnm15034 26351090 PMC4622144

[41] Floria M , Iov D‐E , Tanase DM , Barboi OB , Baroi GL , Burlacu A , Grecu Mihaela , Sascau Radu Andy , Statescu Cristian , Mihai Catalina , Drug Vasile Liviu . Gastro‐esophageal reflux disease and paroxysmal atrial fibrillation ablation. Life 2023;13(5):1107. doi:10.3390/life13051107 37240752 PMC10220808

[42] Sarairah SY , Woodbury B , Methachittiphan N , Tregoning DM , Sridhar AR , Akoum N . Esophageal thermal injury following cryoballoon ablation for atrial fibrillation. JACC Clin Electrophysiol 2020;6(3):262–268. doi:10.1016/j.jacep.2019.10.014 32192675

[43] Coutinho EL , Herbella FAM , Lovato CAV , Patti MG , Schlottmann F , de Paola AAV . Objective evaluation of gastroesophageal reflux disease in patients with paroxysmal atrial fibrillation. World J Surg 2018;42(5):1458–1462. doi:10.1007/s00268-017-4337-4 29134307

