# Supplementary material for: Gastroesophageal reflux disease and risk of atrial fibrillation/flutter: Implications for heart failure progression
Source: ESC Heart Fail. 2025 Nov 9;12(6):4401–9. doi: 10.1002/ehf2.70009 (PMC12719815; doi:10.1002/ehf2.70009)
Supplement: Supplementary file 1 — Table S1. Descriptive details about exposure dataset and outcome dataset. [file EHF2-12-4401-s003.docx]

**Table S1: Descriptive details about exposure dataset and outcome dataset**

| **Exposure or outcome** | **GWAS ID** | **Cases** | **Controls** | **Sample size** | **Participants** |
| --- | --- | --- | --- | --- | --- |
| GERD | ebi-a-GCST90000514 | 129080 | 473524 | 602604 | European |
| Obesity | finn-b-E4_OBESITYNAS | 4,793 | 209,884 | 214677 | European |
| AF/AFL | finn-b-I9_AF | 22068 | 116926 | 138994 | European |
