# Supplementary material for: Gastroesophageal reflux disease and risk of atrial fibrillation/flutter: Implications for heart failure progression
Source: ESC Heart Fail. 2025 Nov 9;12(6):4401–9. doi: 10.1002/ehf2.70009 (PMC12719815; doi:10.1002/ehf2.70009)
Supplement: Supplementary file 2 — Table S2. Summary statistics for the instrumental variables associated with GERD and AF/AFL. [file EHF2-12-4401-s002.docx]

**Table S2: Summary statistics for the instrumental variables associated with GERD and AF/AFL**

| SNPs | Chr | A1 | A2 | GERD | | | | | |  | AF/AFL | | |
| --- | --- | --- | --- | --- | --- | --- | --- | --- | --- | --- | --- | --- | --- |
|  |  |  |  | EAF | Beta | SE | P | R^2^ | F |  | Beta | SE | P |
| rs10010963 | 4 | T | C | 0.616 | -0.027 | 0.005 | 4.92E-08 | 3.44E-04 | 207.51 |  | -0.008 | 0.016 | 6.20E-01 |
| rs1011407 | 2 | G | A | 0.122 | -0.042 | 0.007 | 1.09E-08 | 3.78E-04 | 227.88 |  | -0.017 | 0.027 | 5.31E-01 |
| rs10133111 | 14 | A | G | 0.163 | 0.042 | 0.007 | 1.35E-10 | 4.76E-04 | 287.25 |  | 0.016 | 0.018 | 3.83E-01 |
| rs1021363 | 10 | G | A | 0.642 | -0.031 | 0.005 | 5.10E-10 | 4.48E-04 | 270.06 |  | -0.006 | 0.017 | 7.17E-01 |
| rs10837002 | 11 | G | C | 0.351 | 0.028 | 0.005 | 4.03E-08 | 3.48E-04 | 210.02 |  | 0.010 | 0.016 | 5.35E-01 |
| rs11762636 | 7 | A | C | 0.180 | -0.051 | 0.006 | 1.88E-16 | 7.83E-04 | 472.43 |  | -0.028 | 0.017 | 9.06E-02 |
| rs11953061 | 5 | T | C | 0.339 | 0.028 | 0.005 | 3.10E-08 | 3.55E-04 | 214.20 |  | -0.032 | 0.015 | 3.90E-02 |
| rs12204714 | 6 | T | C | 0.632 | -0.029 | 0.005 | 7.92E-09 | 3.86E-04 | 232.80 |  | 0.006 | 0.015 | 6.98E-01 |
| rs12357321 | 10 | A | G | 0.311 | 0.032 | 0.005 | 1.33E-09 | 4.31E-04 | 259.93 |  | 0.002 | 0.016 | 8.94E-01 |
| rs12453010 | 17 | T | C | 0.395 | 0.030 | 0.005 | 1.75E-09 | 4.21E-04 | 254.06 |  | 0.011 | 0.015 | 4.94E-01 |
| rs12598916 | 16 | G | C | 0.275 | -0.033 | 0.005 | 6.87E-10 | 4.41E-04 | 265.83 |  | -0.028 | 0.017 | 1.00E-01 |
| rs12967855 | 18 | G | A | 0.670 | -0.037 | 0.005 | 1.09E-12 | 5.90E-04 | 355.85 |  | -0.016 | 0.017 | 3.42E-01 |
| rs12997558 | 2 | A | G | 0.359 | 0.028 | 0.005 | 3.04E-08 | 3.56E-04 | 214.63 |  | 0.010 | 0.015 | 5.35E-01 |
| rs13107325 | 4 | T | C | 0.074 | 0.070 | 0.009 | 2.20E-14 | 6.78E-04 | 408.86 |  | -0.162 | 0.064 | 1.21E-02 |
| rs1334297 | 13 | A | G | 0.734 | -0.039 | 0.005 | 1.14E-12 | 5.87E-04 | 354.21 |  | -0.032 | 0.017 | 5.70E-02 |
| rs13409451 | 2 | G | A | 0.392 | -0.028 | 0.005 | 1.93E-08 | 3.66E-04 | 220.69 |  | -0.035 | 0.016 | 2.47E-02 |
| rs1431196 | 18 | G | A | 0.428 | 0.032 | 0.005 | 2.65E-11 | 5.15E-04 | 310.35 |  | 0.013 | 0.015 | 3.89E-01 |
| rs1479405 | 12 | T | C | 0.322 | 0.031 | 0.005 | 9.85E-10 | 4.33E-04 | 260.80 |  | 0.030 | 0.016 | 6.51E-02 |
| rs1510719 | 4 | C | T | 0.383 | -0.039 | 0.005 | 3.84E-15 | 7.15E-04 | 431.12 |  | 0.006 | 0.015 | 7.13E-01 |
| rs1592757 | 5 | C | G | 0.356 | 0.031 | 0.005 | 6.00E-10 | 4.44E-04 | 267.38 |  | 0.020 | 0.016 | 2.01E-01 |
| rs1596747 | 2 | G | A | 0.494 | 0.031 | 0.005 | 1.00E-10 | 4.83E-04 | 291.28 |  | 0.038 | 0.015 | 1.02E-02 |
| rs1716171 | 12 | T | C | 0.790 | 0.038 | 0.006 | 7.83E-11 | 4.89E-04 | 294.92 |  | 0.007 | 0.018 | 6.93E-01 |
| rs17379561 | 1 | T | A | 0.144 | 0.053 | 0.007 | 1.08E-14 | 6.96E-04 | 419.66 |  | 0.017 | 0.018 | 3.26E-01 |
| rs1883842 | 20 | G | T | 0.279 | 0.031 | 0.005 | 9.27E-09 | 3.83E-04 | 230.70 |  | 0.012 | 0.019 | 5.49E-01 |
| rs1937450 | 1 | G | T | 0.538 | 0.032 | 0.005 | 7.07E-11 | 4.96E-04 | 299.01 |  | 0.040 | 0.015 | 7.91E-03 |
| rs2016933 | 3 | G | C | 0.730 | -0.031 | 0.005 | 1.04E-08 | 3.79E-04 | 228.71 |  | 0.029 | 0.017 | 1.01E-01 |
| rs2023878 | 19 | T | C | 0.192 | -0.036 | 0.006 | 3.04E-09 | 4.09E-04 | 246.63 |  | -0.006 | 0.017 | 7.37E-01 |
| rs2043539 | 7 | A | G | 0.419 | 0.027 | 0.005 | 2.24E-08 | 3.60E-04 | 217.19 |  | 0.007 | 0.015 | 6.38E-01 |
| rs215614 | 7 | A | G | 0.630 | -0.033 | 0.005 | 4.08E-11 | 5.03E-04 | 303.48 |  | -0.034 | 0.017 | 3.95E-02 |
| rs2164300 | 4 | T | C | 0.523 | -0.026 | 0.005 | 4.13E-08 | 3.50E-04 | 210.81 |  | -0.011 | 0.015 | 4.77E-01 |
| rs2240326 | 3 | A | G | 0.474 | -0.047 | 0.005 | 1.13E-22 | 1.11E-03 | 669.24 |  | -0.005 | 0.015 | 7.24E-01 |
| rs2396133 | 7 | G | A | 0.475 | 0.029 | 0.005 | 1.11E-09 | 4.30E-04 | 259.11 |  | 0.005 | 0.015 | 7.52E-01 |
| rs2396766 | 7 | A | G | 0.473 | 0.032 | 0.005 | 2.34E-11 | 5.17E-04 | 311.77 |  | 0.026 | 0.015 | 8.31E-02 |
| rs2734839 | 11 | T | C | 0.607 | -0.028 | 0.005 | 8.79E-09 | 3.84E-04 | 231.19 |  | -0.031 | 0.015 | 3.45E-02 |
| rs2744961 | 6 | T | C | 0.358 | 0.029 | 0.005 | 5.81E-09 | 3.92E-04 | 236.41 |  | 0.006 | 0.016 | 7.29E-01 |
| rs2782641 | 1 | A | G | 0.613 | 0.027 | 0.005 | 4.33E-08 | 3.48E-04 | 209.93 |  | -0.019 | 0.015 | 2.21E-01 |
| rs2815749 | 1 | G | A | 0.801 | 0.039 | 0.006 | 1.07E-10 | 4.82E-04 | 290.52 |  | -0.007 | 0.021 | 7.32E-01 |
| rs2834005 | 21 | C | T | 0.315 | 0.030 | 0.005 | 9.42E-09 | 3.81E-04 | 229.47 |  | 0.006 | 0.017 | 7.06E-01 |
| rs2838771 | 21 | C | G | 0.647 | -0.028 | 0.005 | 2.91E-08 | 3.61E-04 | 217.48 |  | 0.004 | 0.016 | 8.08E-01 |
| rs324769 | 12 | T | C | 0.449 | -0.027 | 0.005 | 3.05E-08 | 3.55E-04 | 213.77 |  | 0.008 | 0.015 | 5.82E-01 |
| rs329122 | 5 | A | G | 0.420 | -0.029 | 0.005 | 3.05E-09 | 4.08E-04 | 246.15 |  | -0.004 | 0.015 | 7.67E-01 |
| rs3766823 | 1 | A | G | 0.171 | 0.039 | 0.006 | 7.09E-10 | 4.40E-04 | 265.37 |  | -0.001 | 0.018 | 9.36E-01 |
| rs3793577 | 9 | G | A | 0.538 | 0.027 | 0.005 | 2.49E-08 | 3.63E-04 | 218.94 |  | 0.013 | 0.015 | 3.93E-01 |
| rs3828917 | 6 | T | G | 0.042 | 0.067 | 0.012 | 2.27E-08 | 3.61E-04 | 217.62 |  | -0.063 | 0.058 | 2.84E-01 |
| rs3863241 | 8 | T | C | 0.527 | 0.032 | 0.005 | 1.49E-11 | 5.27E-04 | 317.46 |  | 0.008 | 0.015 | 5.84E-01 |
| rs4300861 | 2 | T | C | 0.382 | 0.031 | 0.005 | 5.43E-10 | 4.45E-04 | 268.53 |  | 0.003 | 0.015 | 8.48E-01 |
| rs4382592 | 9 | G | T | 0.700 | -0.030 | 0.005 | 8.20E-09 | 3.85E-04 | 232.17 |  | 0.029 | 0.017 | 7.65E-02 |
| rs4713692 | 6 | T | C | 0.368 | -0.028 | 0.005 | 3.07E-08 | 3.55E-04 | 213.75 |  | -0.003 | 0.016 | 8.43E-01 |
| rs569356 | 1 | G | A | 0.141 | -0.038 | 0.007 | 4.07E-08 | 3.48E-04 | 209.76 |  | -0.037 | 0.022 | 9.69E-02 |
| rs6711584 | 2 | A | G | 0.452 | 0.032 | 0.005 | 2.66E-11 | 5.15E-04 | 310.73 |  | 0.011 | 0.015 | 4.69E-01 |
| rs6722661 | 2 | A | G | 0.365 | -0.032 | 0.005 | 1.15E-10 | 4.82E-04 | 290.63 |  | -0.013 | 0.016 | 4.18E-01 |
| rs6780459 | 3 | T | A | 0.747 | 0.031 | 0.006 | 3.14E-08 | 3.53E-04 | 212.88 |  | 0.003 | 0.018 | 8.83E-01 |
| rs7032155 | 9 | A | C | 0.592 | 0.028 | 0.005 | 1.63E-08 | 3.72E-04 | 224.27 |  | 0.022 | 0.015 | 1.45E-01 |
| rs7206608 | 16 | G | C | 0.323 | 0.029 | 0.005 | 1.46E-08 | 3.72E-04 | 224.06 |  | 0.017 | 0.016 | 2.84E-01 |
| rs7241572 | 18 | A | G | 0.209 | 0.037 | 0.006 | 9.49E-10 | 4.42E-04 | 266.40 |  | 0.031 | 0.018 | 9.12E-02 |
| rs7527682 | 1 | G | A | 0.537 | -0.027 | 0.005 | 3.13E-08 | 3.54E-04 | 213.42 |  | -0.011 | 0.015 | 4.73E-01 |
| rs7541875 | 1 | G | A | 0.426 | 0.027 | 0.005 | 1.61E-08 | 3.67E-04 | 221.30 |  | 0.006 | 0.015 | 6.96E-01 |
| rs7600261 | 2 | T | C | 0.306 | 0.034 | 0.005 | 9.47E-11 | 4.86E-04 | 292.81 |  | -0.023 | 0.016 | 1.49E-01 |
| rs7612999 | 3 | A | G | 0.245 | 0.031 | 0.006 | 4.90E-08 | 3.45E-04 | 207.96 |  | 0.018 | 0.018 | 3.01E-01 |
| rs761777 | 10 | G | A | 0.254 | 0.035 | 0.006 | 4.71E-10 | 4.52E-04 | 272.50 |  | 0.014 | 0.017 | 3.92E-01 |
| rs7675588 | 4 | A | C | 0.795 | -0.034 | 0.006 | 1.80E-08 | 3.67E-04 | 221.10 |  | -0.023 | 0.020 | 2.59E-01 |
| rs7685686 | 4 | G | A | 0.422 | -0.028 | 0.005 | 1.14E-08 | 3.80E-04 | 229.33 |  | -0.007 | 0.015 | 6.49E-01 |
| rs773109 | 12 | A | G | 0.335 | -0.038 | 0.005 | 8.71E-14 | 6.46E-04 | 389.27 |  | -0.018 | 0.016 | 2.66E-01 |
| rs7942368 | 11 | T | C | 0.215 | -0.034 | 0.006 | 9.54E-09 | 3.89E-04 | 234.52 |  | -0.035 | 0.018 | 5.69E-02 |
| rs903678 | 1 | A | G | 0.339 | 0.028 | 0.005 | 4.89E-08 | 3.45E-04 | 207.98 |  | 0.037 | 0.016 | 2.42E-02 |
| rs903959 | 8 | A | T | 0.399 | 0.029 | 0.005 | 2.99E-09 | 4.08E-04 | 245.95 |  | 0.001 | 0.015 | 9.59E-01 |
| rs9372625 | 6 | A | G | 0.383 | -0.038 | 0.005 | 2.62E-14 | 6.73E-04 | 405.66 |  | -0.011 | 0.016 | 4.93E-01 |
| rs9373363 | 6 | G | A | 0.254 | -0.033 | 0.006 | 4.13E-09 | 4.04E-04 | 243.81 |  | -0.032 | 0.016 | 4.34E-02 |
| rs9396740 | 6 | A | G | 0.249 | -0.031 | 0.006 | 1.47E-08 | 3.71E-04 | 223.49 |  | 0.008 | 0.018 | 6.53E-01 |
| rs942065 | 14 | A | G | 0.634 | 0.031 | 0.005 | 8.45E-10 | 4.38E-04 | 264.34 |  | -0.001 | 0.015 | 9.53E-01 |
| rs9529055 | 13 | A | G | 0.476 | 0.027 | 0.005 | 3.11E-08 | 3.55E-04 | 213.72 |  | -0.025 | 0.015 | 8.80E-02 |
| rs9542729 | 13 | G | C | 0.202 | -0.036 | 0.006 | 1.41E-09 | 4.26E-04 | 256.79 |  | 0.015 | 0.017 | 3.55E-01 |
| rs9615905 | 22 | T | C | 0.458 | 0.028 | 0.005 | 1.21E-08 | 3.77E-04 | 227.43 |  | 0.008 | 0.015 | 5.77E-01 |
| rs9636202 | 19 | A | G | 0.267 | -0.035 | 0.005 | 1.51E-10 | 4.80E-04 | 289.55 |  | -0.020 | 0.018 | 2.73E-01 |
| rs9940128 | 16 | A | G | 0.422 | 0.033 | 0.005 | 8.06E-12 | 5.39E-04 | 325.15 |  | 0.057 | 0.015 | 1.49E-04 |
