# Supplementary material for: Gastroesophageal reflux disease and risk of atrial fibrillation/flutter: Implications for heart failure progression
Source: ESC Heart Fail. 2025 Nov 9;12(6):4401–9. doi: 10.1002/ehf2.70009 (PMC12719815; doi:10.1002/ehf2.70009)
Supplement: Supplementary file 3 — Table S3. MR‐PRESSO test of causal association between Exposure and Outcome. [file EHF2-12-4401-s004.docx]

**Table S3. MR-PRESSO test of causal association between Exposure and Outcome**

| MR-PRESSO test of causal association between GERD and the risk of AF/AFL | | | |
| --- | --- | --- | --- |
| Result one | Main MR results | P value | |
|  | Causal estimate of raw | 6.40E-06 | |
|  | Causal estimate of outlier-corrected | NA | |
| Result two | Outlier SNPs | RSSobs | P value |
|  | NA | 95.83 | 0.077 |
|  |  |  |  |
| MR-PRESSO test of causal association between AF/AFL and the risk of GERD | | | |
| Result one | Main MR results | P value | |
|  | Causal estimate of raw | 3.52E-01 | |
|  | Causal estimate of outlier-corrected | NA | |
| Result two | Outlier SNPs | RSSobs | P value |
|  | NA | 36.42 | 0.023 |
