# Supplementary material for: Gastroesophageal reflux disease and risk of atrial fibrillation/flutter: Implications for heart failure progression
Source: ESC Heart Fail. 2025 Nov 9;12(6):4401–9. doi: 10.1002/ehf2.70009 (PMC12719815; doi:10.1002/ehf2.70009)
Supplement: Supplementary file 4 — Table S4. Summary statistics for the instrumental variables associated with AF/AFL and GERD. [file EHF2-12-4401-s001.docx]

**Table S4: Summary statistics for the instrumental variables associated with AF/AFL and GERD**

| SNPs | Chr | A1 | A2 | GERD | | | | | |  | AF/AFL | | |
| --- | --- | --- | --- | --- | --- | --- | --- | --- | --- | --- | --- | --- | --- |
|  |  |  |  | EAF | Beta | SE | P | R^2^ | F |  | Beta | SE | P |
| rs10507248 | 12 | T | G | 0.698 | 0.103 | 0.016 | 2.29E-10 | 4.43E-03 | 618.50 |  | -0.007 | 0.006 | 0.215 |
| rs1071521 | 1 | G | A | 0.745 | -0.112 | 0.017 | 3.04E-11 | 4.78E-03 | 667.84 |  | -0.003 | 0.006 | 0.630 |
| rs10753933 | 1 | G | T | 0.642 | -0.086 | 0.016 | 2.44E-08 | 3.42E-03 | 476.42 |  | -0.009 | 0.005 | 0.057 |
| rs10883903 | 10 | G | A | 0.235 | 0.113 | 0.017 | 9.12E-11 | 4.55E-03 | 635.01 |  | -0.003 | 0.006 | 0.589 |
| rs12509595 | 4 | C | T | 0.311 | 0.089 | 0.016 | 3.18E-08 | 3.39E-03 | 473.43 |  | -0.008 | 0.005 | 0.149 |
| rs12988307 | 2 | C | T | 0.222 | 0.137 | 0.018 | 1.03E-14 | 6.45E-03 | 902.28 |  | 0.000 | 0.006 | 0.977 |
| rs147790633 | 10 | C | T | 0.146 | -0.131 | 0.021 | 5.03E-10 | 4.28E-03 | 598.04 |  | -0.004 | 0.007 | 0.569 |
| rs17042121 | 4 | G | A | 0.150 | 0.480 | 0.020 | 2.21E-123 | 5.88E-02 | 8690.95 |  | 0.003 | 0.008 | 0.727 |
| rs2209073 | 17 | A | G | 0.632 | -0.086 | 0.015 | 2.00E-08 | 3.42E-03 | 477.64 |  | -0.005 | 0.005 | 0.370 |
| rs34515871 | 1 | T | C | 0.300 | 0.156 | 0.016 | 3.84E-22 | 1.02E-02 | 1432.91 |  | 0.013 | 0.005 | 0.009 |
| rs3807989 | 7 | G | A | 0.572 | 0.128 | 0.015 | 1.75E-17 | 8.01E-03 | 1122.19 |  | 0.005 | 0.005 | 0.266 |
| rs529526 | 5 | C | T | 0.286 | 0.130 | 0.016 | 2.56E-15 | 6.87E-03 | 961.92 |  | 0.016 | 0.005 | 0.002 |
| rs56147946 | 1 | T | C | 0.038 | 0.217 | 0.038 | 1.17E-08 | 3.43E-03 | 478.71 |  | -0.019 | 0.012 | 0.102 |
| rs67329386 | 16 | T | C | 0.226 | 0.174 | 0.018 | 3.33E-23 | 1.06E-02 | 1488.12 |  | 0.005 | 0.006 | 0.450 |
| rs6838973 | 4 | T | C | 0.499 | -0.194 | 0.015 | 2.77E-39 | 1.87E-02 | 2651.71 |  | 0.002 | 0.005 | 0.666 |
| rs75557443 | 11 | T | C | 0.098 | 0.148 | 0.025 | 2.72E-09 | 3.89E-03 | 542.20 |  | -0.001 | 0.008 | 0.910 |
| rs7633500 | 3 | A | G | 0.445 | -0.087 | 0.015 | 4.93E-09 | 3.77E-03 | 526.36 |  | 0.009 | 0.005 | 0.063 |
| rs880315 | 1 | C | T | 0.412 | 0.101 | 0.015 | 1.70E-11 | 4.98E-03 | 695.84 |  | 0.004 | 0.005 | 0.463 |
| rs9825233 | 3 | T | C | 0.588 | 0.106 | 0.015 | 2.42E-12 | 5.40E-03 | 754.88 |  | -0.006 | 0.005 | 0.238 |
